# Supplementary material for: Saccharomyces boulardii (CNCM I-745) improves intestinal damage in sepsis by remodeling intestinal flora structure
Source: Front Cell Infect Microbiol. 2025 Jul 31;15:1602792. doi: 10.3389/fcimb.2025.1602792 (PMC12350329; doi:10.3389/fcimb.2025.1602792)

Venn Diagram

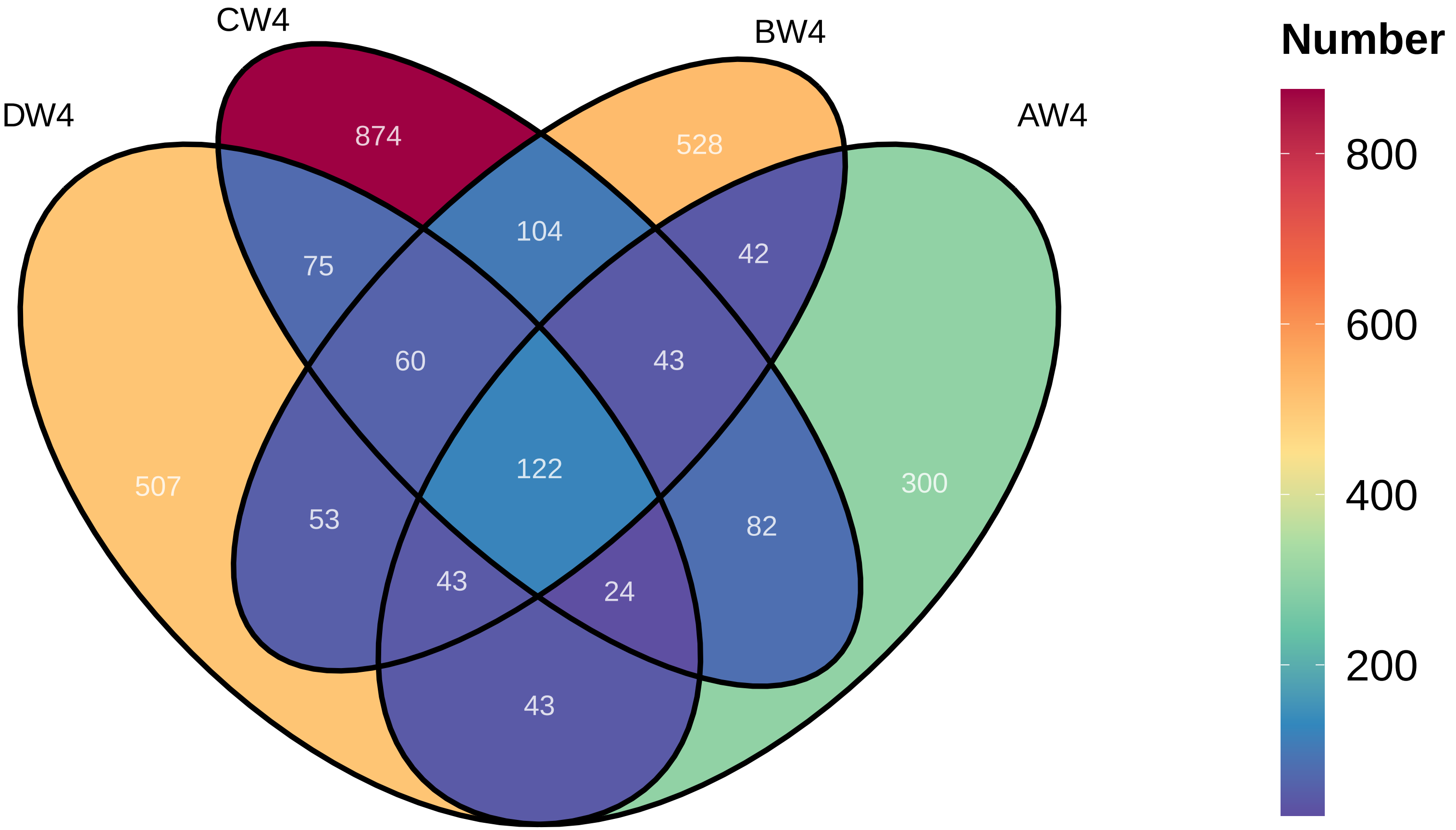

Supplement: Supplementary file 1 [file DataSheet1.zip › summary/summary/3_ASV_profiling/2_venn_diagrams/venn_diagram_Group.DW4_vs_CW4_vs_BW4_vs_AW4.pdf]
